# Intestinal Parasite Infections and Associated Risk Factors among Pre-School Aged Children in Kibera Informal Settlement, Nairobi, Kenya

**DOI:** 10.24248/eahrj.v6i1.683

**Published:** 2022-07

**Authors:** Daniel Njenga, Amos K. Mbugua, Collins Okoyo, Sammy M. Njenga

**Affiliations:** a College of Health Sciences (COHES), Jomo Kenyatta University of Agriculture and Technology (JKUAT), Nairobi, Kenya; b Eastern and Southern Africa Centre of International Parasite Control (ESACIPAC), Kenya Medical Research Institute (KEMRI), Nairobi, Kenya

## Abstract

**Background:**

Infections with intestinal parasites are a major public health problem in children in developing countries like Kenya. School going children are considered at most risk and are included in school-based de-worming program. Less focus is given to pre-school children and information is scarce about intestinal parasitosis among this age group. In this study, we determined the prevalence and intensity of protozoa and helminth infections, and associated risk factors in an informal settlement.

**Methods:**

A community based cross-sectional study was conducted from October 2016 to January 2017 among 406 children aged 2-5 years in Kibera informal settlements in Nairobi County, Kenya. Structured interviewer-administered questionnaire was used to collect sociodemographic information and data on associated factors. Stool samples were examined microscopically using formal ether concentration, iodine wet mounting, modified Ziehl-Neelsen staining, and Kato-Katz methods. Multivariable logistic regression analysis was used to identify risk factors associated with intestinal parasites.

**Results:**

The overall prevalence of any helminth and protozoa infections was 13.1% 53/406) and 22.4% (91/406) respectively. The predominant parasites were *Giardia lamblia* (13.8%), *Ascaris lumbricoides* (11.3%), *Entamoeba histolytica/dispar* (9.4%), *Trichuris trichiura* (3.9%), *Entamoeba coli* (1.5%) and hookworm (0.2%). Prevalence of co-infection with any helminths or protozoan was 2.7%. About 10.8% (44/406) and 20.7% (84/406) children were infected with single species of helminth and protozoan parasites. All helminth infections were light, with a mean intensity of 592 egg per gram. Intensity of any protozoan infections was heavy 62.6% (57/406). Dirt floors in the household (aOR = 2.22, *p = .046*), dirty toilets (aOR = 2.33, *p=.014*), water from communal taps (aOR = 0.27, *p=.019*), parent's education level (aOR=0.27, *p=.032*) and parent's earning (aOR =3.34, *p=.007*) were factors significantly associated with intestinal parasites.

**Conclusion:**

The study found both helminth and protozoan parasites to be prevalent among pre-school aged children in Kibera. Intervention measures including education on the improvement of hygiene and health, socio-economic conditions, sanitation, and provision of safe drinking water could reduce the prevalence of these infections.

## BACKGROUND

Intestinal parasite infections (IPIs) caused by helminths and protozoa are among the most prevalent and persistent infections globally. They constitute a major public health burden worldwide, caused mostly due to faecal contamination of food and water.^1^ Estimates by the World Health Organization (WHO) show that globally, 3.5 billion individuals are affected, and that approximately 450 million people suffer from these infections, with majority being children residing in developing countries.^2,3^ The primary parasites responsible for IPIs are soil-transmitted helminths (STHs) including; roundworm (*Ascaris lumbricoides*), whipworm (*Trichuris trichiura*), and hookworms (*Ancylostoma duodenale* and *Necator americanus*), and pathogenic intestinal protozoa such as *Entamoeba histolytica, Giardia lamblia* and *Cryptosporidium* species.^2,4^

Among helminth infections, ascariasis caused by *A. lumbricoides* is the most prevalent IPIs with approximately 807 to 1,221 million infections globally. Majority of these infections occur in sub-Saharan Africa and East Asia. Hookworms and whipworms (*Trichuris trichiura*) account for 576 to 740 and 604 to 795 million infections, respectively.^4^ The WHO considers STH infection as one of the Neglected Tropical Diseases (NTDs) with the greatest public health concern.^2^

Infections caused by STHs cause low mortality, however, chronic and repeated infection in children can lead to loss of appetite, vomiting, anaemia and vitamin A deficiency, growth retardation, and poor mental function and cognitive development.^5,6^ *A. lumbricoides* and *T. trichiura* are transmitted through the faecal-oral route, by ingestion of infective eggs from soiled hands, water, or food contaminated with human faeces. Hookworm species are transmitted transdermally, when infective larvae present in contaminated soil penetrate the skin.

With regard to intestinal protozoan infections, *G. lamblia* is the most prevalent intestinal protozoan parasite worldwide with approximately 200 million people infected.^7^ *E. histolytica*, the causative agent of amoebiasis, is responsible for an estimated 40,000 to 100,000 deaths annually.^8^ *Cryptosporidium* is a common opportunistic protozoan associated with diarrhoea among children, elderly, and immuno-compromised individuals.^9^ Worldwide, the prevalence of cryptosporidiosis ranges from 1 to 4.5% in developed countries and 3 to 20% in developing countries.^9^ The enteric protozoa pathogens; *E. histolytica, G. lamblia*, and *Cryptosporidium* species, are frequently associated with acute and chronic diarrhoea, malabsorption syndrome, vitamin A deficiency, stunted growth and weight loss and a higher overall risk of mortality in children.^10^ Infection by *E. histolytica, G. lamblia*, and *Cryptosporidium* species occur by ingestion of infective cysts in food, water, or hands contaminated with faeces.

In Kenya, a developing country in Sub-Saharan Africa (SSA), the prevalence of intestinal parasitic infections poses a serious health problem among children. Presently, the Kenyan government is implementing a National school-based deworming program, aimed at reducing the disease burden associated with STHs among school-going children aged 6 to 14 years.^11^ Numerous studies on Intestinal Parasites (IPs) conducted in Kenya largely focus on school-going children and use school-based surveys.^12–14^ Less attention is placed on IPs in pre-school children (below 5 years) resulting in limited evidence of infection burden among this group. In view of this, an important proportion of the childhood population is excluded from existing control programs, which might lead to persistence of these infections whose long-term effects may continue to have negative health effects throughout their lives. To provide an insight on the magnitude of IPIs situation among pre-school children, this study assessed the prevalence of intestinal protozoa and helminth infections and associated risk factors among pre-school age children (2 to 5 years) in selected villages in Kibera informal settlement located in Nairobi County, Kenya.

## MATERIALS AND METHODS

### Study Design and Study Area

A community based cross-sectional survey was conducted among pre-school aged children in the Kibera informal settlements in Nairobi County, Kenya, between October 2016 and January 2017. Kibera is located approximately 5kms Southwest of the capital city, Nairobi. It is situated at an altitude of 1,670m above sea level and lies between latitude 36°47’0”E and longitude 1°19’0”S. Mean annual temperature and relative humidity range from 12°C to 28°C and 32% to 98%, respectively and the average annual rainfall is about 790mm.^15^ Kibera occupies over 250 hectares and is the largest slum in Nairobi with an estimated population of about 200,000 people.^16^ The informal settlement are composed of 14 villages namely; Kianda, Olympic, Soweto West, Gatwekera, Raila, Karanja, Kisumu Ndogo, Makina, Kambi Muru, Mashimoni, Lindi, Laini Saba, Silanga and Soweto East.^15^ Like many urban slum communities, Kibera is characterised by substandard housing conditions, overcrowding, poor sanitation and lack of safe and clean drinking water, which result to unhealthy living conditions that favour intestinal parasite transmission.^15,17,18^

### Study Population and Eligibility Criteria

The study population constituted of 406 preschool children aged between 2 to 5 years residing in Kibera informal settlements. Children whose parents/guardians gave consent to participate in the study were included. Children on antiparasitic treatment, and treated recently for intestinal parasites, children who had diarrhoea at the time of stool sample collection, and whose parents/guardians did not consent to take part in the study were excluded.

### Sample Size Determination and Sampling Technique

Sample size was determined using a single population proportion formula with the following assumptions: where, *n* is the sample size, *z* (1.96) is the standard deviation at a 95% Confidence Interval, *p* is the prevalence of 40.5% from a previous study among preschool children in Kibera, Nairobi,^18^ and *d* is the margin of error (0.05).

Thus, *n* = z^2^ p (1-p)/d^2^.

Then, n = 1.96^2^*0.405 (0.595)/0.0025 = 370.

By considering a 10% non-response rate, the resulting sample size was 406 participants. Multi-stage sampling was used to identify study villages and households. The sampling frame included 14 villages in Kibera informal settlements. 7 out of 14 villages (first stage), were selected using a simple random sampling technique. The names of the 14 villages were written on pieces of paper which were then folded, placed in a bowl, and mixed. The blind-folded study Principal Investigator (PI) selected the desired sample by picking the required number of papers. Each village corresponding to the name chosen was then included in the sample.

In the second sampling stage, 58 households in each of the chosen villages were selected using systematic sampling procedure and these were assigned unique identifiers (i.e., village name abbreviation and household number). Taking advantage of Kenya's government program, the Community Health Strategy, which maintains updated household listings of all households with children aged 5 years and below in each village,^19^ 58 households in each of the chosen villages were selected from the provided lists. The study was performed with collaboration of the Community Health Workers (CHWs) that work under this program in Kibera. Every third household was picked from the list of designated households. In cases where the interviewers did not find an eligible participant in the house, they proceeded to the next house until an eligible participant was found.

Households were the basic sampling unit and only one participant per household was recruited. For households with more than one child in this age category, only one child was considered using the lottery sampling method. 58 stool samples were obtained from enrolled children from each village (58*7), giving a total of 406 stool samples for the study.

### Data Collection and Processing Household Survey

Prior to data and stool sample collection, preliminary meetings were held with CHWs and village heads of the selected villages to explain to them the study's protocol. The data was collected using a structured questionnaire through face-to-face interviews with the children's parent or guardian. The questionnaire was prepared originally in English, translated into Swahili language and then retranslated back to English. The comparison was conducted to check for accuracy and consistency between the two versions of the questionnaire. The CHWs were trained on the questionnaire content, interview method, objectives of the study, and stool sample collection method by the PI. After the training, the study questionnaire was pre-tested on 5% of the total calculated sample size. Inconsistencies and errors identified were corrected accordingly. Questionnaires were administered by 7 CHWs. The PI prudently supervised the data collection process.

A structured questionnaire comprised of 3 parts. The first part included socio-demographic and economic characteristics of the study respondents such as age, gender, duration of residence, parent/guardian educational status, parent/guardian occupation, family income and household conditions. The second part comprised of information on environmental factors such as water source, toilet availability, toilet distance, toilet cleaning mechanisms and garbage disposal. The last part included questions regarding behavioural factors such as hand washing practice before and after using the toilet, the practice of shoe wearing, habits such as sucking the thumb, nail biting, and fingernail trimming as well as knowledge on transmission and prevention of intestinal parasite infections and history of receiving antiparasitic treatment prior to sample collection.

### Collection of Stool Samples

Parents and guardians were adequately instructed on how to obtain an adequate portion of their child's stool. A single stool specimen was collected from each study participant in a clean, dry plastic container labelled with a unique identifier. Each of the specimens was checked for its quantity and labelling. Stool samples from each village were batched and transported in cool boxes by CHWs on the same day to the Centre for Clinical Research (CCR), Kenya Medical Research Institute (KEMRI) for diagnosis. Samples that could not be examined immediately were stored at 4°C and processed within a maximum of 12 hours post-collection.

### Laboratory Processing and Analysis

Laboratory analysis focused on STHs (*A. lumbricoides, T. trichiura*, and hookworm) and protozoa (*G. lamblia, E. histolytica/dispar* and *Cryptosporidium* species) because they are amongst the most prevalent IPs and important contributors to global morbidity and mortality. Evidence of infection was based on the presence of protozoan cysts and oocysts and STH eggs.

### Stool Sample Processing using Kato-Katz Technique

For the diagnosis of STHs, duplicate Kato-Katz thick smears were prepared from each stool sample using 41.7 mg punched plastic templates.^20^ Smear were mounted on slides and covered with malachite green impregnated cellophane. The slides were examined under the microscope at a magnification of ×10. For hookworms, the slides were read within one hour of smear preparation. The smear slides were left overnight to clear for easy visualisation of other helminth eggs. The STHs eggs for each species were counted and recorded separately. The total numbers of eggs were expressed as Eggs Per Gram (EPG) of stool. The mean EPG was calculated to classify the intensity of each STH infection as light, moderate, and heavy infection according to WHO criteria^21^: for Hookworm (light infection: 1-1999 epg, moderate: 2000-3999 epg, heavy: ≥ 4000 epg). Similarly, for *A. lumbricoides* (light infection: 1-4999 epg, moderate: 5000-49999 epg, heavy: ≥ 50000 epg). Intensity of *T. trichiura* (light: 1-999 epg, moderate: 1000-9999 epg, heavy: ≥ 10000 epg).

### Stool Sample Processing using Formal-Ether Concentration Technique

For diagnosis of intestinal protozoans, the remaining portion of stool specimen were concentrated by formol-ether method to increase yield of cysts, oocysts of protozoan parasites. For each specimen, about 1g of stool was transferred into 10mL of 10% formalin solution, thoroughly mixed using applicator stick and sieved through four layers of wet gauze. About 7mL of the sieved suspension was collected in a centrifuge tube. A volume of 3mL of diethyl ether was added and mixed well by shaking for about 1 minute and centrifuged at 3,000 revolutions per minute (rpm) for 1 minute.^22^ The supernatant was decanted, and the sediment processed and examined using iodine wet mounting and modified Ziehl Neelsen (ZN) staining methods.

### Stool sample processing using Iodine wet mount

Iodine mounts were examined to detect and determine intensities of *G. lamblia* and *E. histolytica* cysts in stool. A drop of sediment (20μl) obtained using formol ether concentration method, was placed on a slide, stained with a drop of 1% lugol's iodine, covered with a 22-by 22-mm cover slip and examined microscopically for protozoan cysts at 100× and 400× magnifications, according to WHO protocol.^22^ Infection intensities was determined semi-quantitatively as; (i) negative (0 cysts in the entire sediment); (ii) rare (1-5 cysts per slide); (iii) frequent (1 cyst per observation field of x400); and (iv) very frequent (≥ 1-cyst per observation field of x400), as described by Utzinger et al.^23^ Entamoeba cysts were reported as *E. histolytica/dispar*, since the two species are morphologically identical and cannot be distinguished microscopically.^24^

### Stool Sample Processing using Modified ZN Staining Technique

This method was used for detection and determination of intensities of *Cryptosporidium* species oocysts in stool. Thin smears of sediments (20μl) from the concentration technique were prepared on a slide, air-dried and fixed in methanol for 2 to 3 minutes. The slides were stained with cold carbolfuchsin for about 5 to 10 minutes, decolourised in 1% hydrochloric acid-ethanol solution for 15 to 30 seconds and thoroughly rinsed in clean tap water, then counter stained with 0.25% malachite green for 30 seconds, rinsed well in clean tap water, air-dried, and examined microscopically at 1000x magnification.^22^ The intensities for *Cryptosporidium* species infection was scored semi quantitatively as: negative (0 oocysts), slight (1-5 oocysts), moderate (6-10 oocysts), severe (>10 oocysts), as described by Castro-Hermida et al.^25^

### Quality Control

To ensure the quality of the investigation and results, CHWs were trained for one day on how to collect data and stool samples. Questionnaires were checked for completeness soon after the interviews. Laboratory examinations were carried out by experienced medical laboratory professionals. Stool samples were randomly selected and examined independently by experienced laboratory technologists and their respective results compared. Final decision of discordant slides was reached based on consensus and in consultation with a senior technologist.

### Statistical Analysis

The observed prevalence and intensity of intestinal protozoan, and helminth infections were calculated by gender and village and 95% confidence intervals (CIs) were determined using binomial logistic regression and negative binomial regression respectively, taking into account clustering by village. Comparisons of prevalence by gender and village were performed using Fisher's exact test. The significance of the factors associated with intestinal protozoan, and helminth infections among the children was determined using a multivariable logistic regression model reporting the odds ratios at 95% CI. The choice of the model was based on the log likelihood function. The minimum adequate covariates for multivariable analysis were selected using the forward stepwise variable selection method which selected covariates with a *p-value* less than .300 in the bivariable model.^26^ All statistical analysis was survey set and carried out using STATA version 12.0 (StataCorp, College Station, TX, USA).

### Ethics Approval and Consent to participate

This study was reviewed and approved by the Scientific and Ethics Review Unit (SERU) of KEMRI (SERU Protocol No. 3012). Official permission to conduct field activities was obtained from the Director of Health Services, Nairobi County. Parents/guardians of the children were thoroughly briefed by CHWs about the study objectives during individual house visits, emphasising that participation was voluntary and that withdrawal from the study at any point was permitted even without reason. Signed or thumb-printed informed consent was obtained from the parents/guardians of the children before sample collection began. Data collected from each child and results of laboratory tests were kept confidential and used only for this study. The test results were returned to the parents/guardians and children found positive for pathogenic intestinal parasites were given referral letters for free treatment at the Médecins Sans Frontières (MSF) supported clinics in the Kibera informal settlement.

## RESULTS

### Socio-Demographic Characteristics of Study Participants

The data was collected from 406 preschool children, aged 2 to 5 years, with a mean age of 3.4 years (Standard Deviation 0.9 years) from 7 villages in the Kibera informal settlement. Information on gender was provided for 404 (99.5%) of the children and 51.7% were female. The age of the care givers of the children was between 19 and 69 years with a mean age of 29.0 years (Standard Deviation 6.2 years), the majority of the care givers (88.9%) were mothers of the children, and 6.2% were guardians. All villages were equally represented in the sample at 14.3% (Tables 1 and 2).

**TABLE 1:** Prevalence (%) and Mean Intensity (Epg) of Intestinal Helminth Infections

	n (%) [N=406]	Any helminths		Hookworm		Ascaris lumbricoide		Trichuris trichiura	
		Prevalence% (95%CI); n	Mean Intensity (epg) [95%CI]	Prevalence% (95%CI); n	Mean intensity (epg) [95%CI]	Prevalence% (95%CI); n	Mean intensity (epg) [95%CI]	Prevalence% (95%CI); n	Mean intensity (epg) [95%CI]
Overall	406 (100%)	13.1% (7.6–22.6); n=55	592 (186–1880)	0.2% (0–1.7); n=1	1 (0–1)	11.3% (6.0–21.4); n=46	577 (179–1861)	3.9% (1.8–8.7); n=16	15 (5–47)
**Village**									
Kambi-Muru	58 (14.3%)	15.5% (8.5–28.3); n=9	32 (6–167)	0	0	15.5% (8.5–28.3); n=9	32 (6–167)	0	0
Lindi	58 (14.3%)	17.2% (9.8–30.3); n=10	948 (142–6337)	0	0	15.5% (8.5–28.3); n=9	883 (117–6690)	5.2% (1.7–15.6); n=3	65 (2–1906)
Silanga	58 (14.3%)	12.1% (6.0–24.2); n=7	383 (39–3741)	0	0	6.9% (2.7–17.8); n=4	373 (16–8777)	5.2% (1.7–15.6); n=3	10 (1–189)
Soweto East	58 (14.3%)	31.0% (21.1–45.5); n=18	2549 (668–9722)	17% 0.2–12.0); n=1	1 (0–115)	29.3% (19.7–43.7); n=17	2523 (625–10146) n=7	12.1% (6.0–24.2);	24 (4–160)
Soweto West	58 (14.3%)	8.6% (3.7–19.9); n=5	88 (7–1122)	0	0	6.9% (2.7–17.8); n=4	85 (5–1547)	3.4% (0.9–13.5); n=2	3 (0–80)
Kianda	58 (14.3%)	1.7% (0.2–12.0); n=1	7 (0–1784)	0	0	0	0	1.7% (0.2–12.0); n=1	7 (0–1784)
Laini Saba	58 (14.3%)	5.2% (1.7–15.6); n=3	138 (4–4717)	0	0	5.2% (1.7–15.6); n=3	138 (4–4716)	0	0
**Gender**										
Male	195 (48.3%)	11.3% (7.6–16.7); n=22	412 (113–1512)	0.5%; (0.1–3.6); n=1	0 (0–35)	10.5% (6.8–15.5); n=20	402 (102–1584)	4.1% (2.1–8.1); n=8	11 (2–70)
Female	209 (51.7%)	14.4% (10.3–20.0); n=30	760 (249–2315)	0	0	12.0% 739 (8.3–17.3); n=25	3.8% (214–2559)	20 (3–145) (1.9–7.6); n=8	

N= total sample size, n= total number of individuals, CI = confidence interval and epg = eggs per gram

**TABLE 2:** Prevalence (%) of Protozoan Infections

	n (%) [N=406]	Any protozoa % (95%CI); n	Entamoeba histolytica % (95%CI); n	Entamoeba coli % (95%CI); n	Giardia lamblia % (95%CI); n
Overall	406 (100%)	22.4% (15.7–32.0); n=91	9.4% (6.5–13.5); n=38	1.5% (0.4–5.2); n=6	13.8% (8.2–23.2); n=56
**Village**					
Kambi–Muru	58 (14.3%)	24.1% (15.3–38.1); n=14	12.1% (6.0–24.2); n=7	0	12.1% (6.0–24.2); n=7
Lindi	58 (14.3%)	20.7% (12.5–34.2); n=12	0	0	20.7% (12.5–34.2); n=12
Silanga	58 (14.3%)	27.6% (18.2–41.9); n=16	10.3% (4.8–22.1); n=6	1.7% (0.2–12.0); n=1	19.0% (11.1–32.3); n=11
Soweto East	58 (14.3%)	12.1% (6.0–24.2); n=7	10.3% (4.8–22.1); n=6	0	1.7% (0.2–12.0); n=1
Soweto West	58 (14.3%)	43.1% (32.1–57.9); n=25	13.8% (7.2–26.2); n=8	1.7% (0.2–12.0); n=1	29.3% (19.7–43.7); n=17
Kianda	58 (14.3%)	12.1% (6.0–24.2); n=7	6.9% (2.7–17.8); n=4	0	5.2% (1.7–15.6); n=3
Laini Saba	58 (14.3%)	17.2% (9.8–30.3); n=10	12.1% (6.0–24.2); n=7	6.9% (2.7–17.8); n=4	8.6% (3.7–19.9); n=5
**Gender**					
Male	195 (48.3%)	21.5% (16.5–28.2); n=42	10.3% (6.8–15.5); n=20	2.1% (0.8–5.4); n=4	12.3% (8.5–17.9); n=24
Female	209 (51.7%)	23.4% (18.4–30.0); n=49	8.6% (5.5–13.4); n=18	1.0% (0.2–3.8); n=2	15.3% (11.1–21.1); n=32

N= total sample size, n= total number of individuals. CI = confidence interval

### Prevalence and intensity of Intestinal Helminth and Protozoan Infections

The overall prevalence of intestinal helminth infection was 13.1% (95% CI: 7.6-22.6); n=53. Prevalence of *A. lumbricoides* was highest 11.3% (95% CI: 6.0-21.4); n=46, while low prevalence was recorded for *T. trichiura as* 3.9% (95% CI: 1.8-8.7); n=16, and hookworm 0.2% (95%CI: 0-1.7); n=1. Besides prevalence, the intensity of infection was estimated from the number of eggs per gram (epg) of stool. *A. lumbricoides* 577 epg, (95% CI: 179-1861), *T. trichiura* 15 epg, (95% CI: 5-47) and hookworm 1 epg, (95% CI: 0-1), all had light infections. There were no children with moderate or heavy infections. Both prevalence and mean intensity of all intestinal helminth infections were relatively high among children from Soweto East village at 31.0% (95% CI: 21.1-45.5); n=18 and 2549 epg (95% CI: 668-9722), (Table 1). Overall, 22.4% (95% CI: 15.7-32.0); n=91 of the children were infected with any protozoan parasites, with *G. lamblia* being the most prevalent at 13.8% (95% CI: 8.2-23.2); n=56, followed by *E. histolytica/dispar* 9.4% (95% CI: 6.5-13.5); n=38 and *Entamoeba coli* 1.5% (95% CI: 0.4-5.2); n=6. Infections with any protozoa were very frequent at 62.6% (95% CI: 49.9-78.6); n=57 (Table 2). The prevalence and intensity of all protozoan infections was highest among participants from Soweto West village at 43.1% (95% CI: 32.1-57.9); n=25 and 72.0% (95% CI: 56.4-91.9); n=18 (Table 2 and 3). Majority of *G. lamblia* infections were very frequent at 83.9% (71.7-98.2); n=47, while those for *E. histolytica* were rare 42.1% (95% CI: 29.5-60.1), (Table 3). In this study, co-infection was defined as simultaneous infection with different intestinal parasite species, either helminths or protozoa. The overall prevalence of co-infection with any helminth or protozoan was 2.7% (95% CI: 1.7-4.3); n=11. No child was co-infected with more than three parasite species. Single infection was more frequent with protozoa at 20.7% (95% CI: 14.0-30.5); n=84, compared to helminths at 10.8% (95% CI: 6.9-17.0); n=44 and. Highest numbers of co-infection were observed in male children 3.6% (95% CI: 1.7-7.4); n=7 compared to female children at 1.9% (95% CI: 0.7-5.1); n=4 (Table 4).

**TABLE 3:** Prevalence (%) of Intensity Of Protozoan Infections

	Any protozoa % (95%CI); n			Entamoeba histolytica % (95%CI); n			Entamoeba coli % (95%CI); n			Giardia lamblia % (95%CI); n		
	Rare	Frequent	Very frequent	Rare	Frequent	Very frequent	Rare	Frequent	Very frequent	Rare	Frequent	Very frequent
**Overall**	20.9% (10.0–43.7): n=19	20.9% (15.7–27.8): n=19	62.6% (49.9–78.6): n=57	42.1% (29.5–60.1): n=16	36.8% (25.2–53.9): n=14	31.6% (20.2–49.5): n=12	16.7% (6.3–44.4): n=1	50.0% (28.4–88.0): n=3	83.3% (49.6–99.9): n=5	5.4% (0.7–42.3): n=3	12.3% (8.3–24.5): n=8	83.9% (71.7–98.2): n=47
**Village**												
Kambi-Muru	57.1% (36.3–90.0): n=8	14.3% (4.0–51.5): n=2	28.6% (12.5–65.4): n=4	71.4% (44.7–90.3): n=5	14.3% (2.3–87.7): n=1	14.3% (2.3–87.7): n=1	0	0	0	42.9% (18.2–100.0): n=3	14.3% (2.3–87.7): n=1	42.9% (18.2–79.8): n=3
Lindi	0	16.7% (4.7–59.1) n=2	83.3% (64.7–99.2): n=10	0	0	0	0	0	0	0	16.7% (4.7–59.1): n=2	83.3% (64.7–99.2): n=10
Silanga	6.3% (0.9–41.7): n=1	31.3% (15.1–64.6): n=5	62.5% (42.8–91.4): n=10	16.7% (2.8–99.7): n=1	66.7% (37.9–98.3): n=4	16.7% (2.8–99.7): n=1	0	0	0	0	18.2% (5.2–63.7): n=2	81.8% (61.9–96.4): n=9
Soweto0East	28.6% (8.9–92.2): n=2	28.6% (8.9–92.2): n=2	42.9% (18.2–99.3): n=3	33.3% (10.8–70.3): n=2	33.3% (10.8–70.3): n=2	33.3% (10.8–70.3): n=2	0	0	0	0	0	100% (95.0–100): n=1
Soweto-West	12.0% (4.2–34.7): n=3	16.0% (6.5–39.3): n=4	72.0% (56.4–91.9): n=18	37.5% (15.3–91.7): n=3	37.5% (15.3–91.7): n=3	25.0% (7.5–83.0): n=2	0	0	0	0	59% (0.9–39.4): n=1	94.1% (83.6–99.7) n=16
Kianda	28.6% (8.9–92.2); n=2	14.3% (2.3–87.7); n=1	71.4% (44.7–90.4); n=5	50.0% (18.8–80.7); n=2	25.0% (4.6–91.3); n=1	50.0% (18.9–80.8); n=2	0	0	0	0	0	100% (95.0–100); n=3
Laini-Saba	30.0% (11.6–77.3); n=3	30.0% (11.6–77.3); n=3	70.0% (46.7–96.3); n=7	42.9% (18.2–90.6); n=3	42.9% (18.2–90.6); n=3	57.1% (30.1–98.3); n=4	25.0% (4.6–91.3); n=1	50.0% (18.8–80.7); n=2	100 (95.0–100); n=4	0	40.0% (13.7–80.5); n=2	100% (95.0–100); n=5
**Gender**												
Male	16.7% (8.5–32.8); n=7	23.8% (13.9–40.9); n=10	66.7% (53.8–82.6); n=28	35.0% (19.3–63.5); n=7	40.0% (23.4–68.4); n=8	40.0% (23.4–68.4); n=8	0	75.0% (42.6–98.5); n=3	75.0% (42.6–98.5); n=3	0	16.7% (6.8–40.8); n=4	91.7% (81.3–99.6); n=22
Female	24.5% (15.0–40.0); n=12	18.4% (10.2–33.1); n=9	59.2% (46.9–74.7); n=29	50.0% (31.5–79.4); n=9	33.3% (17.3–64.1); n=6	22.2% (9.4–52.7); n=4	50.0% (12.5–90.6); n=1	0	100% (95.0–100); n=2	9.4 (3.2–27.5); n=3	12.5% (5.0–31.3); n=4	78.1% (65.0–93.8); n=25

n = total number of individuals. CI = confidence interval

**TABLE 4:** Prevalence of the Co-lnfections with Helminths and Protozoans

Co-infections % (95%CI); n	Helminths % (95%CI); n			Protozoans % (95%CI); n			Co-infection with any helminths or protozoan % (95%CI); n
	**One species**	**Two species**	**Three species**	**One species**	**Two species**	**Three species**	
**Overall**	10.8% (6.9–17.0); n=44	2.0% (0.6–6.6); n=8	0.2% (0–1,7); n=1	20.7% (14.0–30.5); n=84	1.2% (0.5–3.3); n=5	0.5% (0.1–3.5); n=2	2.7% (1.7–4.3); n=ll
**Village**							
Kambi–Muru	15.5% (8.5–28.3); n=9	0	0	24.1% (15.3–38.1); n=14	0	0	5.2% (1.7–15.6); n=3
Lindi	13.8% (7.2–26.2); n=8	3.4% (0.9–13.5); n=2	0	20.7% (12.5–34.2); n=12	0	0	1.7% (0.2–12.0); n=1
Silanga	12.1% (6.0–24.2); n=7	0	0	24.1% (15.3–38.1); n=14	3.4% (0.9–13.5); n=2	0	3.4% (0.9–13.5); n=2
Soweto East	20.7% (12.5–34.2); n=12	8.6% (3.7–19.9); n=5	1.7% (0.2–12.0); n=1	12.1% (6.0–24.2); n=7	0	0	3.4% (0.9–13.5); n=2
Soweto West	6.9% (2.8–17.8); n=4	1.7% (0.2–12.0); n=1	0	41.4% (30.5–56.2); n=24	1.7% (0.2–12.0); n=1	0	3.4% (0.9–13.5); n=2
Kianda	1.7% (0.2–12.0); n=1	0	0	12.1% (6.0–24.2); n=7	0	0	1.7% (0.2–12.0); n=1
Laini Saba	5.2% (1.7–15.6); n=3	0	0	10.3% (4.8–22.1); n=6	3.4% (0.9–13.5); n=2	3.4% (0.9–13.5); n=2	0
**Gender**							
Male	8.2% (5.1–13.1); n=16	2.6% (1.1–6.1); n=5	0.5% (0.1–3.6); n=1	19.5% (14.7–25.9); n=38	1.0% (0.3–4.1); n=2	1.0% (0.3–4.1); n=2	3.6% (1.7–7.4); n=7
Female	12.9% (9.1–18.4); n=27	1.4% (0.5–4.4); n=3	0	22.0% (17.1–28.4); n=46	1.4% (0.5–4.4); n=3	0	1.9% (0.7–5.1); n=4

n = total number of individuals, CI = confidence interval

### Factors Associated with Intestinal Parasitic Infections

In this study, significant factors identified as affecting prevalence of IPs were dirt floors in the household, dirty toilets, water from communal taps, parent's education level and parent's earning (*p<.05*). Whereas, factors such as drainage and flooding, gender, parent/guardian marital status, hand washing habit, consistency of wearing shoes, habit of biting fingernails, and having untrimmed fingernails were not associated with IPs infections (Table 5). The likelihood of being infected by helminths was high in children whose parents/guardians had no formal education (aOR = 0.27, 95% CI: 0.08-0.89, *p = .032*), compared to those whose parents/guardians had attained secondary level education (aOR= 0.78, 95% CI: 0.30-2.06, *p=.618*). Similarly, children from families who earned less than Kenya shillings (Ksh) 5000 were more infected with helminth parasites (aOR= 3.34, 95% CI: 1.40-7.96, *p=.007*) and less infected with protozoans (aOR=1.74, 95% CI: 0.88-3.46, *p=.112*) compared with children from families that earned more than Ksh 5000. Likewise, children from houses with dirt floors were at significant risk for any helminth infection (aOR = 2.22, 95% CI:1.01-4.88, *p = .046*) and at non-significant risk for any protozoan infection (aOR = 1.67, 95% CI:0.85-3.29, *p = .135*), compared to children from houses with cemented floors. In addition, children from households that used water from the communal taps were at less risk for any helminth infection (aOR = 0.42, 95% CI: 0.11-1.66, *p=.218*) and at high risk for any protozoan infection (aOR = 0.27, 95% CI: 0.09-0.81, *p=.019*), compared to children from households that buy water from water vendors. Similarly, children whose parents/guardians reported using dirty toilets were at a high significant risk of protozoan infection (aOR = 2.33, 95% CI: 1.19-4.55, *p=.014*) and at non-significant risk for any helminth infections (aOR = 1.52, 95% CI: 0.63-3.69, *p =.352*). Regarding residence, children in Soweto West village (aOR = 3.64, 95% CI: 1.16-11.46, *p =.027*) were at a significantly high risk for protozoan infections, than other villages. Kambi-Muru village was used as the reference category (Table 5).

**TABLE 5:** Multivariable Analysis of Risk Factors Associated With Any Intestinal Helminth and Protozoan Infections

Factors	Risk factors for any helminth infections aOR (95%Q), p-value	Risk factors for any protozoan infections aOR (95%CI), p-value
**Village**		
Kambi-Muru	Reference	
Lindi	1.13 (0.32–4.02), p=0.842	1.12 (0.40–3.12), p=0.835
Silanga	0.29 (0.06–1.36), p=0.116	1.23 (0.38–4.03), p=0.729
Soweto East	6.45 (0.95–43.52), p=0.056	2.55 (0.49–13.19), p=0.264
Soweto West	0.26 (0.05–1.28), p=0.097	3.64 (1.16–11.46), p=0.027^**^
Kianda	0.12 (0.01–1.23), p=0.074	1.12 (0.34–3.66), p=0.849
Laini Saba	1.30 (0.14–11.68), p=0.814	3.50 (0.65–18.80), p=0.144
**Child gender**		
Male vs Female	0.64 (0.31–1.30), p=0.215	0.90 (0.53–1.54), p=0.698
**Child age**		
2 vs 5 years	1.16 (0.31–4.40), p=0.828	0.58 (0.22–1.55), p=0.278
3 vs 5 years	0.74 (0.21–2.63), p=0.642	0.87 (0.35–2.17), p=0.765
4 vs 5 years	2.04 (0.59–7.02), p=0.257	0.83 (0.33–2.05), p=0.683
**Parent's age**		
≤20 vs >40 years	4.67 (0.41–53.70), p=0.216	0.35 (0.06–2.15), p=0.256
21–30 vs >40 years	3.49 (0.21–2.63), p=0.642	0.47 (0.13–1.72), p=0.253
31–40 vs >40 years	1.65 (0.24–11.48), p=0.612	0.43 (0.11–1.64), p=0.219
**Parent's marital status**		
Single vs Married	1.39 (0.54–3.60), p=0.499	0.57 (0.24–1.33), p=0.192
Separated vs Married	0.40 (0.07–2.25), p=0.299	0.90 (0.28–2.90), p=0.860
**Parent's level of education**		
Primary vs None	0.79 (0.31–2.05), p=0.632	1.04 (0.43–2.53), p=0.923
Secondary vs None	0.27 (0.08–0.89), p=0.032^**^	0.78 (0.30–2.06), p=0.618
Post-secondary vs None	0.94 (0.10–9.09), p=0.960	0.34 (0.06–2.06), p=0.242
**Parent's earnings (Ksh)**		
(5,000 – 10,000) vs < 5,000	3.34 (1.40–7.96), p=0.007^**^	1.74 (0.88–3.46), p=0.112
> 10,000 vs < 10,000	4.40 (0.76–25.37), p=0.097	1.27 (0.35–4.59), p=0.720
**Floor type**		
Dirt vs Cement	2.22 (1.01–4.88), p=0.046^**^	1.67 (0.85–3.29), p=0.135
Tile vs Cement	0.89 (0.07–10.81), p=0.924	1.04 (0.13–8.02), p=0.971
**Source of drinking water**		
Borehole vs Communal tap	Omitted	1.71 (0.07–39.18), p=0.736
Water vendor vs Communal tap	0.42 (0.11–1.66), p=0.218	0.27 (0.09–0.81), p=0.019^**^
**Latrine cleanliness**		
Dirty vs Clean	1.52 (0.63–3.69), p=0.352	2.33 (1.19–4.55), p=0.014^**^
**Waste water drainage channel**		
Yes vs No	1.16 (0.43–3.10), p=0.771	1.48 (0.71–3.10), p=0.292
**Flooding during rainy season**		
Yes vs No	0.81 (0.32–2.03), p=0.650	0.87 (0.46–1.68), p=0.687
**Child wash hands after visiting toilet**		
Yes vs No	0.69 (0.20–2.42), p=0.564	0.42 (0.14–1.26), p=0.121
**Child bite nails**		
Yes vs No	1.51 (0.69–3.29), p=0.305	0.63 (0.32–1.23), p=0.178
**Child wear shoes/sandals**		
Yes vs No	8.20 (0.91–73.84), p=0.061	1.25 (0.42–3.69), p=0.684
**Child nails are trimmed**		
Yes vs No	0.74 (0.26–2.17), p=0.589	2.36 (0.72–7.73), p=0.158

aOR- Adjusted odds ratio

** Indicates a significant p-value (<0.05)

## DISCUSSION

This study's findings indicate that protozoa infections were more prevalent than STH infections, which is consistent with a study conducted in Uganda among pre-school children.^27^ Possible reasons could be due to contaminated water and poor sanitary conditions. In this study, children from households that used dirty toilets or used water from the community taps were at significantly high risk of having protozoa infections. Reports from similar studies in urban slum settings have shown that water contaminated with human faeces would be the infection source of the protozoa such as *Entamoeba* species and *G. lamblia.*^28,29^ In contrast, higher rates of infestation with STHs^18^ and protozoans^14^ were previously reported in studies conducted Kenya. Differences in the prevalence of various parasites in this study could be related to sample size, study population, as well as the techniques used for diagnosis.^30^ The predominant helminth parasite was *A. lumbricoides* followed by *T. trichiura* and hookworm respectively, similar to findings from studies conducted in other localities in SSA^31,32^ where *A. lumbricoides* and *T. trichiura* were the most common helminth parasites. According to the WHO classification of infection intensities, *A. lumbricoides, T. trichiura*, and hookworm had light infections. No moderate or heavy infections were recorded in this study. The overall prevalence of STH parasites found in this study was lower than WHO population treatment level^33^ and could be attributed to morbidity control through deworming of school going - children in the study area. The most common protozoan parasite was *G. lamblia* followed by *E. histolytica/dispar*, and commensal *Entamoeba coli* respectively. This finding is consistent with results from similar studies conducted from other SSA countries^27,34-36^, that indicate that *G. lamblia* and *E. histolytica/dispar* infections are more prevalent in under-five children. Most children had very frequent-intensity protozoa infections. Protozoans’ *G. lamblia* and *E. histolytica/dispar* had very frequent and rare intensity infections respectively.

The prevalence and intensity of protozoan infections among children in this study is of concern and deserves consideration in the development of control and prevention policies by Kenya's Ministry of Health. Although modified ZN staining was applied to identify *Cryptosporidium* parasites, no *cryptosporidium* infection was identified in this study. Possible explanations could be due to the selection/choice of the study population and intermittent excretion of cryptosporidium oocysts. The present study investigated asymptomatic children sampled in the community and used a single stool examination. However, this is in contrast to reports on cryptosporidiosis in children aged below 5 years.^27,37^ Regarding co-infections, some of the children harboured multiple species of helminth and protozoa parasites concurrently. This could be due to shared risk factors such as poor sanitation, improper hygiene, and behaviour of participating children. Co-infection as a marker of poor sanitation and poverty is of clinical significance as individuals with multiple parasite species may suffer from multiple morbidities and increased susceptibility to other infections.^38^ Single infection rate was higher for protozoans compared to helminths. This can be attributed to the common route of transmission, i.e., faecal-oral pathway, especially when people do not practice proper personal and environmental hygiene. In addition, no significant difference was observed in intestinal helminth and protozoa infection between male and female children in this study. Possible reason could be that both girls and boys have similar behavioural habits and engage in similar outdoor activities around their households that could expose them to the same sources of infections. However, higher prevalence rates for helminth infections were observed in Soweto East village than other villages. In this study, children whose parents/guardians have no formal education were significantly at risk for helminth infections compared to those with secondary education. Similar findings have been reported in studies among children showing a significant association of infection rate with the education status of the caregiver.^3,39^ Furthermore, this study showed evidence of a significant association of low family income and dirt floors with helminth infections. Previous studies have demonstrated that poor hygienic and sanitary conditions and other factors related to low socioeconomic status facilitate the transmission of IPs.^40^

### Limitations

Due to limited resources, this study relied on a single stool examination for detection of IPs instead of the recommended standard three samples collected in different days.^41^ The overall prevalence of protozoa infection was probably underestimated since formol-ether concentration and iodine mounting methods are - unable to detect trophozoites. In addition, light microscopy was used to detect and identify the amoebic cysts and therefore differentiations of *E. histolytica* from the morphologically identical species *E. dispar* was not done.

## CONCLUSION

This study showed that both helminth and protozoan parasites are prevalent among pre-school aged children in the Kibera informal settlements. Hence, intervention measures including education on personal hygiene and health, provision of safe drinking water, improvement of socioeconomic status, and sanitation should be taken into account to reduce the prevalence of these infections in the study area. Also, further studies using larger sample size and molecular tools should be conducted to determine the prevalence and intensities of IPIs in the area.

## References

[1] Houweling TA, Karim-Kos HE, Kulik MC, Stolk WA, Haagsma JA, Lenk EJ, et al. Socioeconomic inequalities in neglected tropical diseases: a systematic review. PLoS neglected tropical diseases. 2016;10(5): e0004546. 27171166 10.1371/journal.pntd.0004546 PMC4865383

[2] Hotez PJ, Fenwick A, Savioli L, Molyneux DH. Rescuing the bottom billion through control of neglected tropical diseases. The Lancet. 2009;373(9674):1570–1575. doi: 10.1016/S0140-6736(09)60233-6. 19410718

[3] Mehraj V, Hatcher J, Akhtar S, Rafique G, Beg MA. Prevalence and factors associated with intestinal parasitic infection among children in an urban slum of Karachi. PloS one. 2008;3(11): e3680. doi: 10.1371/journal.pone.0003680. PMC2577067 18997865

[4] Bethony J, Brooker S, Albonico M, Geiger SM, Loukas A, Diemert D, et al. Soil-transmitted helminth infections: ascariasis, trichuriasis, and hookworm. The lancet. 2006;367(9521):1521–1532. doi: 10.1016/S0140-6736(06)6865. 16679166

[5] Stephenson LS, Latham MC, Ottesen EA. Malnutrition and parasitic helminth infections. Parasitology. 2000;121(S1): S23–S38. 11386688 10.1017/s0031182000006491

[6] Crompton DWT, Nesheim MC. Nutritional impact of intestinal helminthiasis during the human life cycle. Annual review of nutrition. 2002;22(1):35–59. 10.1146/annurev.nutr.22.120501.134539 12055337

[7] Ivanov AI. Giardia and giardiasis. Bulgarian Journal of Veterinary Medicine. 2010;13(2).

[8] Ximénez C, Morán P, Rojas L, Valadez A, Gómez A. Reassessment of the epidemiology of amebiasis: state of the art. Infection, Genetics and Evolution. 2009;9(6):1023–1032. 10.1016/j.meegid.2009.06.008 19540361

[9] Rossle NF, Latif B. Cryptosporidiosis as threatening health problem: a review. Asian Pacific Journal of Tropical Biomedicine. 2013;3(11):916–924.

[10] Fletcher SM, Stark D, Harkness J, Ellis J. Enteric protozoa in the developed world: a public health perspective. Clinical microbiology reviews. 2012;25(3):420–449. 22763633 10.1128/CMR.05038-11 PMC3416492

[11] Kenya National School Based Deworming Programme Year 4 Results (2015-2016). http://www.thiswormyworld.org/tumikia-project.

[12] Ngonjo TW, Kihara JH, Gicheru M, Wanzala P, Njenga SM, Mwandawiro CS. Prevalence and intensity of intestinal parasites in school age children in Thika District, Kenya. African Journal of Health Sciences. 2012;21(3–4):153–160.

[13] Ngonjo T, Okoyo C, Andove J, Simiyu E, Lelo AE, Kabiru E, et al. Current status of soil-transmitted helminths among school children in kakamega county, western kenya. Journal of parasitology research. 2016. 10.1155/2016/7680124 PMC4971323 27525108

[14] Njambi E, Magu D, Masaku J, Okoyo C, Njenga SM. Prevalence of Intestinal Parasitic Infections and Associated Water, Sanitation, and Hygiene Risk Factors among School Children in Mwea Irrigation Scheme, Kirinyaga County, Kenya. Journal of Tropical Medicine. 2020;2020. 10.1155/2020/3974156 PMC7238387 32454837

[15] Mutisya E, Yarime M. Understanding the grassroots dynamics of slums in Nairobi: the dilemma of Kibera informal settlements. Int Trans J Eng Manag Appl Sci Technol. 2011;2(2):197–213.

[16] Statistics KNB of. The 2009 Kenya population and housing census. Vol. 1. Kenya National Bureau of Statistics; 2010.

[17] Mbae CK, Nokes DJ, Mulinge E, Nyambura J, Waruru A, Kariuki S. Intestinal parasitic infections in children presenting with diarrhoea in outpatient and inpatient settings in an informal settlement of Nairobi, Kenya. BMC Infectious Diseases. 2013;13(1):243. 23705776 10.1186/1471-2334-13-243 PMC3673844

[18] Davis SM, Worrell CM, Wiegand RE, Odero KO, Suchdev PS, Ruth LJ, et al. Soil-transmitted helminths in pre-school-aged and school-aged children in an urban slum: a cross-sectional study of prevalence, distribution, and associated exposures. The American journal of tropical medicine and hygiene. 2014;91(5):1002–1010. doi: 10.4269/ajtmh.14-0060. 25157123 PMC4228865

[19] Health M of. Taking the Kenya essential package for health to the community: a strategy for the delivery of level one services. Reversing the Trends-The Second National Health Sector Strategic Plan of Kenya. Health Sector Reform Secretariat of the Republic of Kenya Nairobi, Kenya; 2006. i–50 p.

[20] Katz N, Chaves A, Pellegrino J. A simple device for quantitative stool thick-smear technique in schistosomiasis mansoni. Rev Inst Med Trop Sao Paulo. 1972;14(6):397–400. 4675644

[21] WHO. Prevention and control of schistosomiasis and soil-transmitted helminthiasis: report of a WHO expert committee. World Health Organization; 2002. 12592987

[22] WHO. Basic laboratory methods in medical parasitology. World Health Organization; 1991.

[23] Utzinger J, Botero-Kleiven S, Castelli F, Chiodini PL, Edwards H, Köhler N, et al. Microscopic diagnosis of sodium acetate-acetic acid-formalin-fixed stool samples for helminths and intestinal protozoa: a comparison among European reference laboratories. Clinical microbiology and infection. 2010;16(3):267–273. 19456836 10.1111/j.1469-0691.2009.02782.x

[24] Gonin P, Trudel L. Detection and differentiation of Entamoeba histolytica and Entamoeba dispar isolates in clinical samples by PCR and enzyme-linked immunosorbent assay. J Clin Microbiol 2003. 41: 237–241. 12517854 10.1128/JCM.41.1.237-241.2003 PMC149615

[25] Castro-Hermida JA, González-Losada YA, Ares-Mazás E. Prevalence of and risk factors involved in the spread of neonatal bovine cryptosporidiosis in Galicia (NW Spain). Veterinary Parasitology. 2002;106(1):1–10. 11992706 10.1016/S0304-4017(02)00036-5 PMC7125682

[26] Hosmer Jr DW, Lemeshow S, Sturdivant RX. Applied logistic regression. Vol. 398. John Wiley & Sons; 2013.

[27] Buzigi E, Uganda K. Prevalence of intestinal parasites, and its association with severe acute malnutrition related diarrhoea. J Biol Agric Healthcare. 2015;5(2).

[28] Garbossa G, Pía Buyayisqui M, Geffner L, Lopez Arias L, de la Fournière S, Haedo AS, et al. Social and environmental health determinants and their relationship with parasitic diseases in asymptomatic children from a shantytown in Buenos Aires, Argentina. Pathogens and global health. 2013;107(3):141–152. 23683369 10.1179/2047773213Y.0000000087 PMC4003592

[29] Gutierrez-Jimenez J, Torres-Sanchez MG, Fajardo-Martinez LP, Schlie-Guzman MA, Luna-Cazares LM, Gonzalez-Esquinca AR, et al. Malnutrition and the presence of intestinal parasites in children from the poorest municipalities of Mexico. The Journal of Infection in Developing Countries. 2013;7(10):741–747. 24129627 10.3855/jidc.2990

[30] Knopp S, Mgeni AF, Khamis IS, Steinmann P, Stothard JR, Rollinson D, et al. Diagnosis of soil-transmitted helminths in the era of preventive chemotherapy: effect of multiple stool sampling and use of different diagnostic techniques. PLoS Negl Trop Dis 2008;2(11): e331 10.1371/journal.pntd.0000331 18982057 PMC2570799

[31] Aiemjoy K, Gebresillasie S, Stoller NE, Shiferaw A, Tadesse Z, Chanyalew M, et al. Epidemiology of soil-transmitted helminth and intestinal protozoan infections in preschool-aged children in the Amhara region of Ethiopia. The American journal of tropical medicine and hygiene. 2017;96(4):866–872. 28167597 10.4269/ajtmh.16-0800 PMC5392634

[32] Nyakang'o LN, Shivairo RS, Muleke CI, Mokua DO. Soil Transmitted Helminths Prevalence among Pre-School Age Children in Elburgon Municipality, Kenya. African Journal of Science and Research. 2015, (4)6:01–04. Available Online: http://ajsr.rstpublishers.com/

[33] WHO. Guideline: preventive chemotherapy to control soil-transmitted helminth infections in at-risk population groups. World Health Organization; 2017. 29578660

[34] Mulatu G, Zeynudin A, Zemene E, Debalke S, Beyene G. Intestinal parasitic infections among children under five years of age presenting with diarrhoeal diseases to two public health facilities in Hawassa, South Ethiopia. Infectious diseases of poverty. 2015;4(1):49. 26530964 10.1186/s40249-015-0081-x PMC4632267

[35] Tine RC, Faye B, Ndour CT, Sylla K, Sow D, Ndiaye M, et al. Parasitic infections among children under five years in Senegal: prevalence and effect on anaemia and nutritional status. International Scholarly Research Notices. 2013;2013. 10.5402/2013/272701 PMC4890897 27335851

[36] Obala AA, Simiyu CJ, Odhiambo DO, Nanyu V, Chege P, Downing R, et al. Webuye health and demographic surveillance systems baseline survey of soil-transmitted helminths and intestinal protozoa among children up to five years. Journal of Tropical Medicine. 2013;2013. 10.1155/2013/734562 PMC3600298 23533444

[37] Gatei W, Wamae CN, Mbae C, Waruru A, Mulinge E, Waithera T, et al. Cryptosporidiosis: prevalence, genotype analysis, and symptoms associated with infections in children in Kenya. The American journal of tropical medicine and hygiene. 2006;75(1):78–82. 16837712

[38] Steinmann P, Du Z-W, Wang L-B, Wang X-Z, Jiang J-Y, Li L-H, et al. Extensive multiparasitism in a village of Yunnan province, People's Republic of China, revealed by a suite of diagnostic methods. The American journal of tropical medicine and hygiene. 2008;78(5):760–769. 18458311

[39] Zemene T, Shiferaw MB. Prevalence of intestinal parasitic infections in children under the age of 5 years attending the Debre Birhan referral hospital, North Shoa, Ethiopia. BMC research notes. 2018;11(1):58. 29357917 10.1186/s13104-018-3166-3 PMC5778703

[40] Nobre LN, Silva RV, Macedo MS, Teixeira RA, Lamounier JA, Franceschini SC. Risk factors for intestinal parasitic infections in preschoolers in a low socio-economic area, Diamantina, Brazil. Pathogens and global health. 2013;107(2):103–106. 23683337 10.1179/2047773213Y.0000000075 PMC4001485

[41] Tarafder MR, Carabin H, Joseph L, Balolong E Jr, Olveda R, McGarvey ST. Estimating the sensitivity and specificity of Kato-Katz stool examination technique for detection of hookworms, Ascaris lumbricoides and Trichuris trichiura infections in humans in the absence of a ‘gold standard’. International journal for parasitology. 2010;40(4):399–404. 19772859 10.1016/j.ijpara.2009.09.003 PMC2829363

